# The Evils of the Contract System

**Published:** 1911-09

**Authors:** 


### SOCIETY PROCEEDINGS

### The Evils of the Contract System

**Report of the Committee Appointed by the Medical Society of the County of Erie to Investigate the Inethical Contract Practices, Submitted at the Regular Meeting of the Society, June 19, 1911**

THE committee appointed to investigate contract work, wishes to report the result of its investigation up to date, and the remedy which it deemed proper that this society should adopt in order to eradicate this evil. We had not proceeded very far in our investigation, before we became aware of the fact that "something" was "rotten in the state of Denmark." We found this evil to be so widespread as to necessitate careful and close analysis, making it impossible for the committee to hand in a complete report at this meeting. From the data which came under our observation, we estimate conservatively that, at least a hundred and fifty thousand people in this city alone, are entitled to professional services under inethical contracts. I say that our work is incomplete, made so by the magnitude of the evil, and the length of time at our disposal in which to cover it. But in justice to this society, we cannot offer any report without giving a specimen of the despicable, penny contracts held by many of the physicians of Buffalo. The following section relative to the duties of the physician taken from the By-laws of an organisation known as the Fraternal Order of Eagles, is self-explanatory.

SECTION 1. It shall be the duty of the Aerie physician to attend, prescribe for, and perform such surgical work as may be necessary, on all members of the Aerie in good standing and their respective families, also all visiting members and their families without extra charge, except in cases of confinement and primary venereal or chronic diseases, or disabilities existing at the time the member made application for membership.

There are two other organisations which offer contracts similar to the above that came under our notice. These organisations are known as The Loyal Order of Moose, and The Fraternal Order of Orioles. All these organisations exist throughout the country. We found that there are a number of other fraternal insurance organisations which offer contracts of a like nature operating in this city. Among these are The American Order of Foresters, The Order of Red Men, The Macabees and various other societies with peculiar appellations. We found that certain liability insurance companies have physicians under contract to care for their policy holders in case of injury. These physicians are paid a miserable pittance, primarily, to coöperate with the insurance companies against the poor, unfortunate who knowingly gets into the web which the greedy, cunning, and malicious spider has so craftily and slyly woven around him. This insectivorous professional cooperates with the liability insurance company at the expense of his innocent and trusting patient to prevent liability. This, in my opinion, is an offence which should be punishable by law.

We found that a number of the large corporations of this city have fostered a movement of their employees to form associations which are supported by regular weekly assessments, primarily, for their own benefit, and, secondarily, for the benefit of their employees. The employee is the actual supporter of this association while the employer receives the greater part of the benefit. By this arrangement, the employer undergoes no inconvenience. The association supports itself. It hires a physician not only to care for its members when injured while at work, but also arranges to have the physician care for any of them for any physical disturbance which may arise during the time of the contract. In some cases they have hired the physician at a regular monthly salary to care not only for themselves, but for their families as well. They have had no difficulty in securing the services of a physician to engage in this admirable, lofty, and sublime work. Of course, the corporation sees to it that one friendly to its interests secures the position. It is absolutely necessary for the corporation to secure the services of an "honorable man" to perform the duties of this high office. He must work for the interest of the corporation and against the interests of the employees. Whenever there is an injury to any of the employees, the actual supporters of the physician, he must have the welfare of the corporation at heart, and do his best to prevent litigation. What a fine clock-wheel arrangement this is! —the corporation works its employees, the employees work the doctor, the doctor works the corporation, and they all work one another.

We found that practically the entire Italian male population of Buffalo is entitled to medical services under cheap contract, and that the doctors holding these contracts, in some cases, are caring for the families at cut-rates, and a few are giving medicine free of charge. This system has grown from bad to worse. As the field of competition increases, the younger physicians who are from year to year graduating from medicine, are taking up the work more cheaply than their predecessors. What is the result of this mad competition? The physician has thrown off the dignity of his profession and has stooped down to the acceptance of a mere pittance as compensation for his professional services. Of course, the number of patients he must call upon is great, and he cannot, therefore, render to each individual the medical attention required, for he must "Be up and doing," as he must utilise every minute to the best of his personal advantage. Under such a system, there is no incentive to do good work. The people do not realise their danger, and something must be done to educate them to the fact that, far from gaining any salutary advantage, they are committing the greatest folly. The doctors of Italian origin who are holding these contracts realise their mistake, and have given the committee to understand that they would only be too willing to give up their contracts were it not for the fact that they fear that some of the American doctors would only too readily take up the work which they should abandon. Such is their plight, and, if these men make the sacrifice of their penurious positions, this society must do all in its power to prevent the niggard and avaricious medicaster from taking their place. Among the Polish population, the same system is in vogue, but we are not prepared to state the extent of it in this report.

Another phase of this work was brought to our attention. We learned that a large number of the department stores and other business places have associations which contract with the physician in the same manner. Indeed, this system has become so widespread that even a number of the labor organisations have adopted it as "a good thing." In the same manner, and at the same price, they hire the same kind of a doctor, undoubtedly unconscious of the fact that they are catering to a scab.

A certain institution which advertises itself as a hospital, engages in wholesale contracts for an infinitesimal amount, to care for its policy holders who may be individuals or families for any illness of any nature whatsoever. This institution has a dispensary where colored solutions under alphabetical labels are dispensed by an undergraduate. We have learned of still another method of securing "The almighty dollar." We learned that a certain Doctor Richardson, whoever he may be, and twenty other misleading physicians of the United States have prepared a very grandiose and elaborate work which they are handing out at the rate of ten dollars per copy, and charging two dollars extra which entitles the holders of the certificate to free consultation for a period of two years. There is a coöperative plan of procedure between the local representative agent of this erudite organisation and a certain leading drug store which enables them to most thoroughly complete the chain of their depredations. While this very idea smacks of the rankest quackery, yet, it is no worse than some of the other cheap contracts held by supposedly regular men. The only difference between them is that one is direct while the other is an indirect mode of procedure.

Another form of contract considered by your committee, is one which shall tax the efforts of that committee to obtain evidence. It was mentioned that some physicians are in the habit of contracting with their patients prior to their illnesses for sums varying from ten to one hundred dollars annually. If such practice is in vogue, it must be most heartily condemned. This gambling game deserves the severest criticism. The physician who practises it is unscrupulous and mercenary. It is an imposition upon society, and, like all other inethical contracts, must be stigmatised with the utmost opprobrium.

We found that each railroad entering Buffalo has its surgeon under annual contract to care for its employees, or any one except a trespasser who may happen to be injured. We do not know the salary paid to these surgeons, but we understand that it is comparatively small for the services required. The action of the committee in attacking the contract system must not be misunderstood. We do not care whether the physician is overpaid or underpaid. We are attacking it, because the principle underlying it all is inethical, unjust, and, in every case, injurious to the profession in general. The surgeon who holds a railroad contract, holds it with the proviso that he coöperate with the railroad and work against the interests of the poor unfortunate who happens to come under his charge. This is, of course, a good thing for the railroad, a nice thing for the surgeon, since it nets him a yearly salary, but a very bad thing for the public. We found that, undoubtedly, this particular form of contract is the grandparent of the rest. In justice to our sense of fairness, we could not possibly take any action against the small offenders without attacking these polished violators of the ethical laws who are seemingly responsible for all the others.

We believe that the above covers concisely the whole of the contract system. The causes of these existing evils were offered by the committee, and are as follows:

1. Lack of knowledge of the ethics of medicine.

2. Professional jealousy.

3. Indifference of the older practitioners.

4. The example set by some of the older practitioners.

5. Overcrowding of the profession.

6. The tolerance of this state of affairs by all medical societies.

7. The commercialism of the age. The professional struggle not only for the "Almighty dollar" but also for the mite(y) cent.

8. The chief and absolute cause of all, however, we found to be the complete disorganisation of the medical body. This last cause which is the absolute, and the cause a priori is the one to which we must give our attention, namely, the disorganisation of the medical body.

This evil has been growing insidiously in all its various forms for some time, and has been passed unnoticed by the powers which should have throttled it in its infancy. It may be that the older members of the profession have been swollen with pseudo pride, deeming such consideration beneath their dignity, or it may be that they have had no idea of the extent or character of this pernicious evil. Be that as it may, our investigation has disclosed a state of affairs tolerated or disregarded by the medical fraternity which should never have been permitted to exist. When the true state of affairs was brought to the notice of your committee, some were in favor of adopting extreme measures, but the more conservative element prudently advised the adoption of persuasive measures. The first action of the committee was to send out return postcards to every member of this society. As each member received one of these cards, you are all aware of its contents. In the short interval of ten days, two hundred and twenty-five cards were returned—all save three of which were in favor of this movement. The three cards against it bore no signatures. At the subsequent meeting of the committee, such happy returns as this showed us that the profession was alive, and, from the remarks on some of the cards, heartily willing to coöperate with the committee. The cards against this movement were without signatures, so that, whatever opposition there is to it, comes from those who have not the courage of their convictions. A second notice was sent to all those members who had not returned the card, reminding them that they had either overlooked or misplaced it, and up to the present date, we have three hundred and twenty members of this society pledged to support this movement. We are aware of some few men who are opposed to it, but only one of them has had the courage to sign himself against it. The committee charitably refrains from mentioning his name.

In the short time that this committee has had to act, it is sorry to say that it cannot offer a more complete report. Your committee shall not spare its efforts until every man is located who has the audacity to stand for such a system as has been brought to your notice. While almost two-thirds of the members are pledged to this movement, there yet remain about two hundred who have not stated their position, and the committee shall continue its investigation until every member has taken a definite stand. We believe that after every one who has not answered has been interviewed, there will be very few in the opposition. As the committee has been very conservative in the measure it proposes to adopt, it hopes that it will be heartily approved by this society. Many radical suggestions were offered, but, after much deliberation, it resolved to adopt the following:

*Resolved,* That the proposed amendment, if adopted, shall not apply to present contracts, but shall prohibit any extension or renewal of the same—except by action of the society, at a regular meeting, upon request of the interested physician.

Add a section to chapter II of the Constitution and By-laws to be known as section 13, and to read as follows: No one shall become a member of this society or continue as such who engages in contract work, unless it be governmental in character, but this shall not prohibit an agreement for a particular case nor apply to examinations for an adequate fee.

We further recommend: 1. That the president appoint from time to time at his own discretion, special committees to use their influence on any man who continues this inethical practice whether he be a member of this society or not.

2. That a special committee be appointed to confer with the membership committee to devise ways and means to get all desirable men in this society.

3. We recommend that the proper authorities of this Society bring this matter to the attention of the State Society for the purpose of gaining its support.

4. That this society meet more frequently, that its members become more united, and that self-interests be put aside for the common good of all.

We feel that the above resolution, amendment, and recommendations are not too radical. The various faddists, quacks, pseudo scientists, and charlatans, are the sins of our neglect. Are we going to commit more sins of omission? The evils dealt with in this report and practised by the supposedly regular members of the profession, should not be tolerated any longer. We must separate the inethical from the ethical practitioner. We must ostracise him as we have the advertising quack. Nay, we must do more. We must use antagonistic measures if persuasion fails to convince him of his error. The methods of antagonising him have been discussed by the committee, but it is not our purpose to speak of them in this report. We address ourselves so strongly because we feel sure that after all the cards have been returned, there will be very few offenders left. To these men we offer fellowship not only in this society, but through it, also to the society of the state and nation. The membership of this society consists of practically all the physicians of Erie County. Many of the men who do not belong to this society are those who have graduated from medicine within the last few years. and most of these can be easily induced to become members.

The above is the situation which is now confronting us. It is high time for us to separate the sheep from the goats. If medicine is a profession, if it stands for something more than mere commercialism, if it possesses every quality that is honorable and noble, then, we should do nothing to degrade it. We should rather raise it until it has reached the climax of ethics, and its standard has become the highest obtainable. But this can never be accomplished by suffering the contaminating presence of men who engage in degrading work to remain within this or any other medical body. If there be any set of men who wish to continue this debasing practice, let them be separated from those who have enough principle and honor to deem such work beneath the dignity of the medical profession. We therefore recommend that this society use every possible effective measure to bring this about, and purge itself of all those who are unworthy to be regarded as its members. We have not been guided by animosity nor actuated by malice in arriving at our conclusion. We have no feeling of resentment toward any man. We have consistently considered big and little offenders alike. We believe in the utmost charity to all, for, even your committee, has among its members men who are or have been violators. Yet, in justice to our profession, besmeared in the mire of commercialism, we have been compelled to suggest the proposed amendment. With the confidence of honesty, we submit it for your consideration, knowing that the urgency of the situation demands every honorable man's support.

Edward E. Haley, Chairman; Albert T. Lytle, Charles R. Borzilleri, Harry R. Trick, Francis M. O'Gorman, Francis E. Fronczak, Lesser Kauffman, Arthur R. Gibson, Edward E. Koehler, Franklin C. Gram, Arthur C. Schaefer, Harry N. Feltes, Edward Clark, Marshall Clinton, Charles A. Wall, Hugh J. McGee, A. L. Benedict, Marcel Hartwig, Patrick J. Hurley, Henry J. Doll, Wilhelm Brauns, Edmund P. Reimann, Jacob W. Bayliss, William J. O'Donnell, Frederick Frisch.

